# Potential Vitamin E Signaling Mediators in Skeletal Muscle

**DOI:** 10.3390/antiox13111383

**Published:** 2024-11-13

**Authors:** Elisabetta Meacci, Antony Chirco, Mercedes Garcia-Gil

**Affiliations:** 1 Department of Experimental and clinical Biomedical Sciences “Mario Serio”, University of Florence, 50134 Firenze, Italy; 2 Interuniversity Institute of Myology, University of Florence, 50134 Firenze, Italy; 3 Department of Biology, Unit of Physiology, University of Pisa, Via S. Zeno 31, 56127 Pisa, Italy; mercedes.garcia@unipi.it

**Keywords:** skeletal muscle, vitamin E, sphingolipids, myokines, antioxidant action, sarcopenia, microgravity

## Abstract

Vitamin E (Vit E) deficiency studies underline the relevance of this vitamin in skeletal muscle (SkM) homeostasis. The knowledge of the effectors and modulators of Vit E action in SkM cells is limited, especially in aging and chronic diseases characterized by a decline in musculoskeletal health. Vit E comprises eight fat-soluble compounds grouped into tocopherols and tocotrienols, which share the basic chemical structure but show different biological properties and potentials to prevent diseases. Vit E has antioxidant and non-antioxidant activities and both favorable and adverse effects depending on the specific conditions and tissues. In this review, we focus on the actual knowledge of Vit E forms in SkM functions and new potential signaling effectors (i.e., bioactive sphingolipids and myokines). The possible advantages of Vit E supplementation in counteracting SkM dysfunctions in sarcopenia and under microgravity will also be discussed.

## 1. Introduction

**Figure 1:**
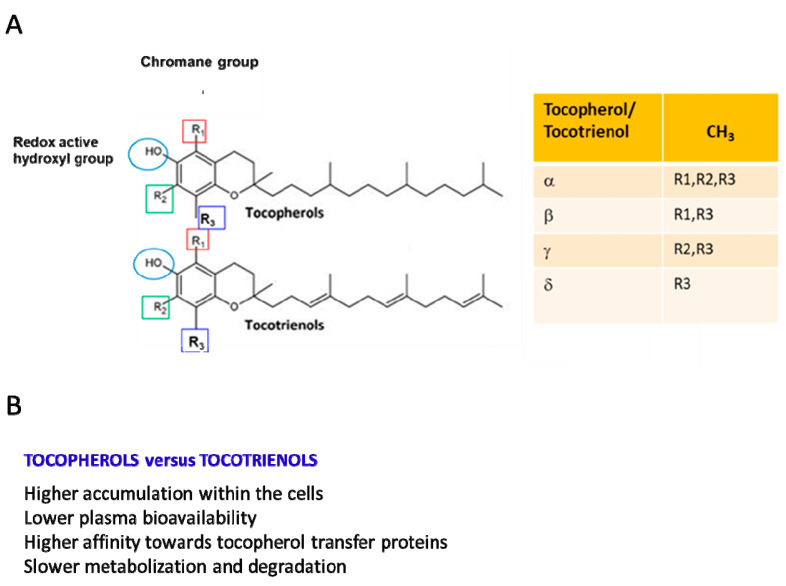
(**A**) Stereochemical structures of tocotrienols and tocopherols. The four isoforms of both tocopherols and tocotrienols differ in the degree and position of methyl groups on the chromanol ring: the α-isomers are trimethylated, the β- and γ-isomers are dimethylated, and the δ-isomers are monomethylated. (**B**) Most relevant biological differences between tocotrienols and tocopherols.

Vitamin E (Vit E), a nutrient found in vegetal oils, was discovered as an antioxidant fat-soluble vitamin involved in the control of reproductive functions and, thus, initially named “anti-sterility factor” [1]. The Vit E family consists of eight hydrophobic compounds that have a similar chemical structure [1] and are grouped into tocopherols and tocotrienols (named α-, β-, γ-, and δ-tocopherol and α-, β-, γ-, and δ-tocotrienol). Tocopherols and tocotrienols are composed of a chromanol ring, which is linked to an isoprenoid side chain at the C2 position. The isoprenoid chain is saturated in tocopherols and unsaturated in tocotrienols (Figure 1A [1,2,3]). Tocopherols and tocotrienols show different biological properties (Figure 1B) [1,2,3].

Vit E is found in plant-based oils (sunflower and soybean oil), nuts, almonds, seeds, fruits, and vegetables, and the major dietary sources of tocopherols are vegetable oils and cottonseed [2]. In particular, depending on the diet, α- δ-, and γ-tocopherol are the predominant forms. For example, α-tocopherol is abundant in the European diet because of the high consumption of olive and sunflower oil, whereas in the US diet, γ-tocopherol is about three times more abundant because of the widespread use in this country of corn and soybean oil enriched in γ-tocopherol. β-tocopherol is present only in small amounts [1,4,5,6]. Food and drug administration recommendations indicate a dietary intake of Vit E based only on α-tocopherol consumption. The recommended dietary allowance for both adult men and women is 15 mg (35 µmol)/day of α-tocopherol [7]. Due to their lipid-solubility, tocopherols and tocotrienols introduced through the diet are easily incorporated into micelles, then absorbed in the small intestine and, without any specific selectivity for tocopherols and tocotrienols, transported to the liver [1,8]. Alpha-tocopherol is selectively retained in liver cells due to its binding to the α-tocopherol transfer protein (α-TTP) [3,5,9], a cytosolic protein that prevents α-tocopherol metabolism. Alpha-TTP presents a very low affinity to β-tocopherol, γ-tocopherol, and δ-tocopherol (Figure 1B). Thus, the non-α-tocopherol forms are metabolized via a cytochrome P450-dependent pathway [9]. From liver cells, α-tocopherol reaches all other tissues. Generally, tocotrienols have higher cellular uptake than tocopherols, with some variations among α-, β-, γ-, and δ-analogs (Figure 1B).

In mammalian plasma, α-tocopherol is the prevalent form of Vit E (22–34 µM), while the others are found at much lower concentrations. In particular, γ-tocopherol and β-tocopherol levels are approximately 10-fold lower than those of α-tocopherol and δ-tocopherol (≈50-fold lower), ranging from 0.3 to 0.8 µM [10]. The three tocotrienols are 100-fold less concentrated than α-tocopherol (<1 µM). Notably, when 800 IU of dl-α-tocopheryl acetate was supplemented for 30 days in humans, the plasma concentration of α-tocopherol increased by 300%, and that of γ-tocopherol decreased by 74%. Similarly, the gastrocnemius muscle showed a significant 53% increase in α-tocopherol and a 37% decrease in γ-tocopherol [10,11,12]. In contrast with α-tocopherol, γ-tocopherol is more bioavailable in tissues than in plasma [11]. In human tissues, γ-tocopherol is the second most abundant Vit E form, and its concentration is higher in skin, adipose, and muscle tissues than in plasma. The concentration of α-tocopherol in SkM is 155 nmol/g, and that of γ-tocopherol is equivalent (107 nmol/g). γ-tocopherol is metabolized faster than α-tocopherol [12,13,14,15]. A faster turnover has also been observed for tocotrienols compared with tocopherols [13,14] (Figure 1B).

Over time, the definition of Vit E deficiency has been based on circulating and tissue α-tocopherol concentrations, the susceptibility of erythrocytes to hemolysis, and peripheral neuropathy [14]. Vit E deficiency is mainly linked to food insecurity, dietary habits, and disorders [15]. Premature newborns of a very low birth weight (<1500 g) are at increased risk of Vit E deficiency because this vitamin crosses the placenta in small amounts, leading to a very low reserve in newborns [15]. With age, the risk of Vit E deficiency decreases since Vit E is present in breast milk and commercial formulas. In adults, disorders that impair absorption (such as certain liver and pancreas disorders and cystic fibrosis) can reduce the absorption of Vit E. In addition, genetic impairments, such as mutations in α-TTP, which cause impaired fat metabolism or errors in lipoprotein production, can determine Vit E deficiency [16]. Vit E deficiency can also be secondary to abetalipoproteinemia and can cause poor transmission of nerve impulses and muscle weakness [17].

Presently, there is a scientific debate on Vit E's nomenclature. Azzi and colleagues proposed to limit the term Vit E only to RRR-α-tocopherol (the R configuration at positions 2, 4, and 8 in the chromanol ring) and not to other tocopherols and tocotrienols [18] since only α-tocopherol is used to protect against Vit E deficiency in humans. Traber (2014) and Eggersdorfer et al. (2024) [14,19] have reported that all α-tocopherols with 2R configurations possess the requirements to be considered Vit E. Other authors do not agree with the restriction of the name since the molecular mechanisms underlying vitamin E deficiency and the prevention or reversion of this disease by vitamin E are not yet completely clarified. Noguchi and Niki [20] have suggested collecting more data on the functions and molecular mechanisms of action of tocochromanols before any nomenclature revision.

In the present review, we point out the relevance of the biochemical properties of tocopherols and tocotrienols as antioxidants, membrane stabilizers, and signal transduction modulators in SkM. Myokines and the bioactive sphingolipid sphingosine 1-phosphate (S1P), both released from SkM cells and modulators of biological processes in this tissue, as well as in others, will be suggested as potential mediators of Vit E signaling. Finally, we present the putative advantage of Vit E supplementation as a strategy in SkM dysfunctions associated with aging and under microgravity.

## 2. Tocopherols and Tocotrienols in Skeletal Muscle

### 2.1. Skeletal Muscle Pathophysiology

SkM tissue constitutes approximately 45-50% of the total body mass. Its prevalent function lies in sustaining movement and posture [21]. However, in the last decade, its role has been extended to the control of thermogenesis and metabolism of other tissues due to its ability to release myokines, cytokines, and other peptides during SkM contraction [22,23]. SkM is responsive to many stimuli, such as exercise, diet, and inflammation, and changes in SkM's mass, function, and strength occur across the life course [23]. Extensive exercise leads to reactive oxygen species (ROS), which can determine the oxidative damage and mitochondrial dysfunctions of SkM fibers [24,25]. Unexpectedly, prolonged muscle inactivity, such as limb immobilization, also promotes an increase in ROS, which leads to fiber atrophy [26]. Oxidative stress due to ROS accumulation can cause an unbalance between protein synthesis and degradation by promoting the expression of the ubiquitin ligase muscle RING-finger protein-1 (MurF-1) and atrogin-1, and the activation of the ubiquitin–proteasome pathway [27], the protease cascade, and the autophagic process [28,29]. The physiological decline in SkM mass and function, named sarcopenia, is characterized by SkM atrophy and changes in muscle fiber composition by the shift from fast to slow fibers [30,31]. Annual loss in muscle mass and strength is gender–dependent and can reach a rate of 0.64–0.70% per year in women and 0.80–0.98% per year in men [28]. Several biological and molecular changes can contribute to SkM atrophy associated with aging, such as a decline in neuromuscular function, hormonal deficits, chronic inflammation, and loss of mitochondrial function and neuromuscular remodeling [32]. In addition, the accumulation of ROS and nitrotyrosine in aging is usually linked to nuclear and mitochondrial DNA damages, which contribute to the irreversible loss of SkM fibers through apoptotic pathways. In older adults, SkM fibers, especially type II fibers, become thinner and shorter in association with a loss of muscle strength [30,33]. SkM mass loss can also occur during muscular disuse and in pathological circumstances such as cancer and diabetes [34,35,36].

### 2.2. Vit E's Effects on Skeletal Muscle Aging

**Figure 2:**
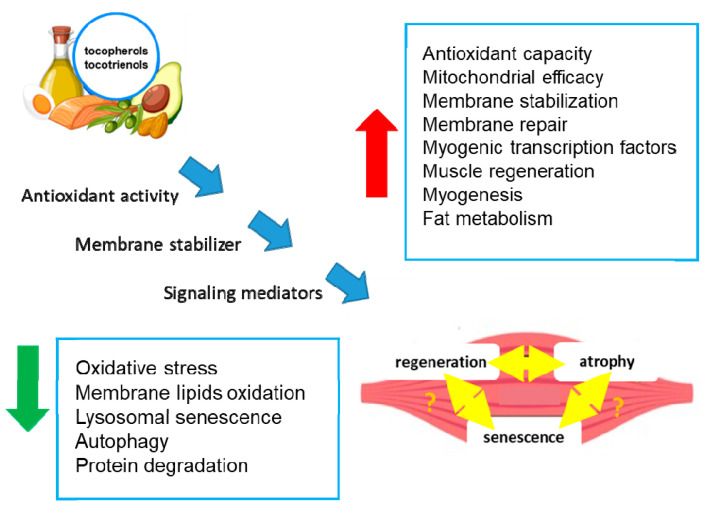
Effects of tocopherols and tocotrienols on skeletal muscle. The figure shows the potential molecular mechanisms (blue arrows) by which tocopherols and tocotrienols act in SkM cells, leading to the modulation of biochemical processes (blue boxes) and tissue regeneration or atrophy. The double yellow arrows indicate an unknown relationship. Green arrow: decrease; red arrow: increase.

Numerous studies have investigated the effects of antioxidants on SkM performance, and recent reports have underlined the important role of tocopherols and tocotrienols in SkM health and their positive effects (Figure 2) [37,38,39,40].

Vit E can delay SkM dysfunctions through several mechanisms, including antioxidant activity, membrane and mitochondria stabilization, and the promotion of SkM precursor cell proliferation [37,38,39,40] (Figure 2). In particular, several in vitro and in vivo studies have underlined the role of Vit E in the control of SkM tissue homeostasis. For example, tocopherols, extracted from chestnut flour, limit cell atrophy and favor myotube survival [41]. Moreover, Vit E contributes to membrane repair in myoblasts damaged by laser, in addition to counteracting oxidative damage [38]. Treatment with Trolox, a Vit E analog, restores the proliferative and regenerative capability of senescent satellite cells [34,37]. It has also been reported that a tocotrienol-rich fraction can limit the induction of lysosomal senescence, ameliorate replicative senescence alterations, and promote myogenesis by regulating myogenic regulatory factor expression [42] (Figure 2).

Human trials that have specifically evaluated the effects of Vit E on musculoskeletal disorders associated with age remain scarce and have mostly focused on α-tocopherols. It has been reported that reduced levels of α-tocopherols are correlated with a higher risk of chronic diseases (i.e., diabetes and cardiovascular disease), suggesting that a high level of tocopherols may be critical for ensuring longevity in healthy centenarians [43,44,45]. Moreover, a reduced risk of hip fractures, incident frailty, SkM and bone loss, and cognitive decline can be associated with α-tocopherol intake (i.e., 15 mg/die) [44,45]. Although there is no evidence that the aging process impairs Vit E absorption or utilization, a positive relationship between the presence of optimal levels of circulating tocopherols and adequate antioxidant activity and immune response has been observed in centenarians [7]. Furthermore, supplementation with whey protein, Vit E, and vitamin D can significantly preserve SkM mass and recover strength in sarcopenic subjects [46]. Another study positively linked daily Vit E supplementation to knee extension strength and physical performance [47]. Furthermore, supplementation with vitamin C and Vit E could lead to a reduction in SkM damage after downhill runs in moderately trained males [48]. In humans, a lower content of Vit E in the serum can promote muscle dysfunctions and muscle deterioration in older subjects, likely due to the higher composition of type I fibers, which are more susceptible to damage than type II fibers [49,50]. Indeed, it has been shown that type I fibers have a high oxidative metabolism and utilize more α-tocopherol to prevent oxidative damage [10]. It has also been reported that RRR α-tocopherol supplementation (150 IU, corresponding to 100 mg) for 12 weeks can counteract SkM damage by protecting against oxidative stress in old men (65–80 years) [51]. Meanwhile, some reports indicate negative results for high-dose Vit E (235 mg) and vitamin C (1000 mg) supplementation in training adaptation [52].

A tocotrienol-rich fraction, composed of 26.67% α-tocotrienol, 4.29% β-tocotrienol, 32.60% γ-tocotrienol, 15.53% δ-tocotrienol, and 20.81% α-tocopherol, was reported to reverse senescence in a stress-induced pre-senescence model of myoblasts [53] to restore the regenerative capacity of the human senescent satellite cells and to ameliorate defense mechanisms against senescence-associated oxidative stress [53,54] (Figure 2). In particular, the supplementation with the tocotrienol-rich fraction potentiated the activity of superoxide dismutase and catalase and reduced glutathione peroxidase activity [54]. Notably, in another preclinical study involving Sprague Dawley rats of different ages undergoing forced swimming exercise, supplementation with a tocotrienol-rich fraction (60 mg/kg/day) for 3 months was associated with a significant decrease in age-related lipid metabolism, an increase in amino acid metabolism, enhanced endurance, and reduced oxidative stress [55] (Figure 2). These results suggest that a tocotrienol-rich fraction may have a positive impact on SkM performance and oxidative stress, which are relevant factors in the context of sarcopenia. Therefore, foods rich in tocopherols can be of help in achieving healthy aging by avoiding Vit E reduction that could contribute to high inflammation and oxidation, the cause of most age-related pathologies [56]. The role of regular exercise in maintaining SkM functions has been extensively discussed [47,48] and is outside the scope of this review. However, exercise has beneficial effects by reducing oxidative stress, not only in SkM but also in all tissues, helping delay aging [57].

### 2.3. Skeletal Muscle Alterations in Microgravity

Prolonged space flights are responsible for reduced mechanical stimulation of SkM, leading to SkM mass wasting [58,59]. Moreover, long-term spaceflight in microgravity (30 days onboard Bion M1) impaired SkM regenerative processes [60], which appeared to not be sufficient to counteract SkM damage [61]. In particular, microgravity exposure during parabolic flights could limit the fusion between membranes and membrane repair [62]. In mice, microgravity effects were evaluated in the International Space Station during the Mice Drawer System program. SkM atrophy occurred in the soleus muscle and not in the extensor digitorum longus muscle and indistinctly in all fiber types to different extents when the animals were evaluated after 91 days compared with 20 days of spaceflight. Most of these changes were qualitatively very similar to those in humans (i.e., a partial shift in muscle fibers toward the glycolytic phenotype) [63,64,65]. Notably, studies performed on twenty healthy male volunteers evaluated after 60 days of simulated microgravity showed that the levels of serum and salivary Vit E concentrations were significantly decreased [66]. Therefore, we can speculate that nutritional interventions and Vit E supplementation during spaceflight could be one strategy of success for the prevention of SkM mass and strength loss. In the NASA twin study, physiological, proteomic, and metabolomic data were collected over 25 months of human spaceflight [67]. The study led to the conclusion that it is crucial to counterbalance the collateral effects of microgravity in future missions. The new findings in this field will be helpful in the health protection of astronauts, and they will also contribute to a better understanding of the molecular mechanisms at the basis of SkM physiology and age-associated SkM dysfunctions.

## 3. Tocopherols and Tocotrienols: Effects on Skeletal Muscle Membranes

The localization and dynamic behavior of tocopherols and tocotrienols in intracellular membranes are essential for the mechanism of action of these molecules. Three main features of Vit E are crucial for its function in the bilayer structures: (1) the depth of penetration of the chroman head group; (2) the orientation of the whole molecule with respect to the bulk phospholipids; and (3) the dynamics of tocopherol’s movement in the bilayers. Because of their unsaturated isoprenoid tail compared with the phytyl tail of tocopherol, tocotrienols show a better ability to diffuse through the membrane phospholipid bilayer [68] and penetrate into tissues that have saturated fatty acid layers, such as the brain and liver. It is known that α-tocopherol can spontaneously associate with polyunsaturated fatty acids, and the chromanol methyl groups are likely responsible for this association [69]. The effects of α-tocopherol on membranes are more complicated than expected since they also seem to be dependent on the phospholipid composition, often unique for membranes of different cell types [70]. Therefore, in the last decades, many studies have focused more on the functional influence of Vit E, especially α-tocopherol, on membrane properties rather than its antioxidant biological action [68]. Therefore, we will briefly discuss the antioxidant and non-antioxidant activity of both tocopherols and tocotrienols in SkM, and we will focus more on their recently described roles as modulators of membrane properties and signaling.

### Antioxidant Activity, Membrane Stabilization, and Membrane Signaling Activity

The mechanistic details of the antioxidant action of tocopherols and tocotrienols are well established in various tissues and nicely revised in [19,68] and are outside the scope of this review. The understanding of the molecular mechanisms and identification of new mediators that regulate the balance between the beneficial or harmful effects of ROS are trending in SkM biology. In SkM, the three most relevant sources of ROS have been reported: mitochondria, nicotinamide adenine dinucleotide phosphate oxidase enzymes (NOX), and xanthine oxidase [71].

Intermediate filaments, such as desmin and vimentin, possess a conserved cysteine residue, which is the target for oxidative and electrophilic modifications, leading to functional dysregulation [72]. Mutations in the desmin gene (i.e., C333S mutation) lead to the accumulation of granulo-filamentous desmin-positive aggregates and myopathy, which can be reduced by antioxidant treatment by up to 75% [72]. Other myofilament proteins, including actin, myosin heavy chain, and troponin C, can be oxidized, and their function impaired, by long-term exposure to oxidants [73,74,75].

ROS are crucial regulators of SkM responses, such as increased glucose uptake and mitochondrial biogenesis, which lead to SkM adaptations [76]. Most of these adaptations in SkM may be potentiated or blunted by antioxidants [77]. In fact, antioxidants, which disturb ROS signaling, can lead to the disruption of exercise training’s beneficial health effects, as confirmed by a recently published meta-analysis of randomized controlled trials [78,79].

The beneficial effect of Vit E is observed in membranes damaged by eccentric exercise but not by other forms of stress, such as aerobic exercise, where the sarcolemma damage is essentially due to metabolic dysfunctions [38,80,81]. During endurance training, Vit E supplementation (400 IU/day) prevents some negative SKM effects [10,82]. Meydani et al. [10] reported that Vit E provides protection against exercise-induced oxidative injury, while Beaton et al. [83] did not find protective effects on SkM damage. Silva et al. [80] demonstrated that α-tocopherol supplementation (800 IU/day of d-α-tocopherol acetate) decreased muscular and oxidative damage but not the inflammatory response induced by eccentric contraction. Another study reported by Yfanti et al. [84] indicated that supplementation with vitamins C and E (1 g of ascorbic acid and 400 IU of d-α-tocopherol daily) had no effect either on the lipid profile or insulin sensitivity during chronic eccentric exercise. Beneficial effects of tocotrienols on SkM contractile damage after repeated and prolonged contractions have also been observed [85]. The discrepancy among these data can be due to the differences in training protocols and the vitamin dosages that were used.

Tocopherol is capable of affecting membrane stabilization by decreasing membrane fluidity, as demonstrated for the first time in intestinal brush-border membranes [68,86]. This effect appears to be a particular property of α-tocopherol but not of β-, γ-, or δ-tocopherol, suggesting the importance of the number of chroman methyl groups and the ability to form van der Waals interactions with membrane phospholipids. The changes in the membrane fluidity and stability contribute to the re-organization of membrane components [68,86,87].

The presence of tocopherols in the double layer of biological membranes contributes to the formation of specific domains, such as cholesterol and sphingolipid-enriched microdomains (lipid rafts), which can, in turn, result in stabilizing or destabilizing effects other than the promotion of specific signaling pathways. In SkM, the stability of both the sarcolemma and myofibrils is crucial for the generation and transmission of force, cell motility, and signaling molecules, which anchor to the extracellular matrix. Although different actors, such as intermediate filaments and the dystrophin–glycoprotein complex, contribute to sarcolemma stabilization, eccentric muscle contractions can lead to physical SkM weakness and tissue degeneration [88].

Plasma membrane disruption, formation, and repair are impaired in mdx mice, a model of Duchene’s muscular dystrophy, and in patients with muscular dystrophy, a genetic disease caused by mutations in dystrophin [89]. Lipid-directed antioxidant activity, such as that of tocopherols, can contribute to both in vitro and in vivo membrane repair [38,83]. In patients with Duchene’s muscular dystrophy, the damage of SkM fibers is also, in part, due to the very low regenerative capacity of stem cells [90]. Therefore, any actions finalized to guarantee stem cell functions may have a therapeutic significance for Duchene’s muscular dystrophy [90]. Stem cells respond to treatment with antioxidants by staying in an undifferentiated functional status [91]. In mdx mice, the supplementation with a tocotrienol-rich fraction can favor stem cell proliferation and differentiation [80].

By regulating membrane fluidity and stability, Vit E can affect the kinetics of interactions between proteins, contributing to the activation of downstream signaling pathways (i.e., p44/42 mitogen-activated kinase (MAPK), PI3K/Akt/mTOR, JAK/STAT, NF-κB nuclear respiratory factor2 (NRF2), and PGC-1α) [92,93]. Understanding the biological functions mediated by α-tocopherol and other Vit E components as cell signaling modulators is of interest, especially when considering various cell types and pathological conditions. Differences in Vit E uptake, intracellular transport, metabolism, and biological effects appear to be cell-specific [68,94]. For example, Vit E can induce or prevent apoptosis [92,95,96]. Antioxidants have the potential to defer disuse muscle atrophy, but the mechanisms involved in this protection are not fully elucidated [97]. Servais et al. [98] reported that the protective effect of α-tocopherol acetate might be due to its ability to modulate muscle proteolysis-related genes (MurF-1 and atrogin-1) and caspases and μ-calpain genes rather than its antioxidant function [98]. Other studies suggested the involvement of mitochondria, demonstrating that tocopherol and the mitochondrial-targeted antioxidant SS-31 could localize in mitochondria and, in turn, exert their actions by protecting muscles against inactivity-induced atrophy [99].

## 4. Potential Mediators of Tocopherols and Tocotrienols’ Action in Skeletal Muscle

### 4.1. Myokines

The molecular and cellular events modulated by Vit E in protecting SkM from aging and disease-associated alterations are still partially unknown, especially those regarding SkM as endocrine tissue. Recent studies show that in response to exercise and, preferentially, during resistance training, SkM produces and releases bioactive molecules, which contribute to crosstalk between the SkM itself and other organs, such as the brain, adipose tissue, and bone through autocrine, paracrine, or endocrine pathways [100,101,102]. The first described exercise-induced factor was IL-6, successively named myokine [103,104,105]. During contraction, SkM is able to secrete hundreds of peptides (more than 650). In the SkM itself, myokines can control cell proliferation, differentiation, tissue regeneration [104,106], and mass [107,108]. The SkM secretome is also involved in immunological responses and anti-inflammatory and anti-cancer processes [107]. The role of myokines in tumor progression is outside the scope of this review. However, it is worth noting that crosstalk between SkM and tumors exists, and there is the possibility of counteracting tumor progression by regulating SkM endocrine function [109]. Studies on the regulation of myokines by Vit E are scarce.

In working muscle, IL-6 levels increase up to 100-fold compared with the pre-exercise baseline [110]. Notably, IL-6 can act as a pro-inflammatory and anti-inflammatory cytokine when it is released by SkM during exercise [111,112]. In addition, IL-6 can inhibit the production of TNF-α and IL-1β and promote other anti-inflammatory cytokines [112]. In SkM, IL-6 controls the activation of satellite cells and potentiates protein synthesis in myotubes via the mTOR signaling cascade, leading to tissue hypertrophy. In fact, the genetic loss of IL-6 results in impaired muscle mass in vivo [113]. IL-6 also mediates the effects of repetitive eccentric contraction, leading to an increase in the stem cell number and fusion of muscle fibers [114].

A correlation between Vit E and IL-6 secretion and inflammation has been demonstrated in several tissues [115]. Notably, a meta-analysis of randomized clinical trials involving 2102 individuals aged from 20 to 70 years demonstrated the beneficial effects of α-tocopherol supplementation on subclinical inflammation through the variation in IL-6 levels [116]. There is little and indirect information regarding Vit E and IL-6 in SkM. It has been reported that dietary vitamin supplementation (mixed tocopherols, flavonoids, and docosahexaenoate) reduced the concentration of inflammatory mediators, such as C-reactive protein and IL-6, in the SkM of untrained males after eccentric exercise [117]. Long-term daily multi-vitamin supplementation can also reduce pro-inflammatory responses and the increase in the IL-6/IL-10 ratio after total knee arthroplasty [118]. The acute inflammatory response to LPS, the consequent IL-6 production, and the decrease in grip strength were exacerbated in α-TTP-null mice [119]. Hypoxia provokes oxidative stress and inflammation, and exercise under this condition leads to additional stress, increasing IL-6, TNF-α, interleukin-1 receptor antagonist, and IL-10 levels immediately after exercise. Supplementation with Vit E (an acute dose of Vit E of 250 mg) counteracts these effects [120]. A meta-analysis showed that dietary Vit E supplementation (300 to 1318 IU per day) significantly reduced biomarkers related to exercise-induced SkM damage and oxidative stress, in particular, IL-6 [115]. However, another systematic review and meta-analysis concluded that supplementation with Vit E had no effect on IL-6 levels after physical exercise in healthy participants [121]. The dosage of pro-inflammatory markers’ protein levels in the SkM of diabetic mice showed that IL-6 and TFN-α levels were lower in the specimens that had been supplemented with a tocotrienol-rich fraction compared with the untreated control group. The same, however, did not apply to monocyte chemoattractant protein-1 levels [122].

Myostatin, a member of the transforming growth factor-β superfamily, is mainly expressed in SkM [123] and is a negative regulator of SkM growth, both during embryogenesis and in adulthood [124]. In particular, the inhibition of satellite cell proliferation and differentiation and muscle fiber protein synthesis occurs in the presence of myostatin, and increased levels of myostatin are significantly associated with SkM-wasting diseases, such as cancer cachexia and sarcopenia [125]. Myostatin is also a crucial regulator of energy metabolism in myoblasts [126]. Growing evidence supports the role of this myokine in obesity, insulin resistance, and cardiovascular and chronic kidney disease [127]. Several pharmacological approaches leading to the reduction in myostatin levels have been suggested as a potential target for dystrophy and other myopathies [128]. Myostatin content was negatively correlated with Vit E intake in prepubertal healthy children having omnivorous diets [129]. Treatment with a tocotrienol-rich fraction (26.67% α-tocotrienol, 4.29% β-tocotrienol, 32.60% γ-tocotrienol, 15.53% δ-tocotrienol, and 20.81% α-tocopherol) promotes the downregulation of myostatin expression in myoblasts [54].

Irisin, a member of the PGC-1α superfamily, is one of the most recent myokines described to date [105]. As with other myokines, irisin is produced by the cleavage of the transmembrane protein FNDC5 and secreted by SkM during physical exercise [130]. Irisin is a short-lived molecule involved in many physiological and pathological conditions [131]. For example, it affects the process of browning of white adipose tissue, thus contributing to thermogenesis and energy metabolism [132]. Regarding SkM, irisin influences the activation/proliferation of satellite cells and myoblasts, promotes myoblast fusion, thus contributing to muscle growth, and can improve regeneration after tissue injury [23,130]. Direct evidence of the regulation of irisin production by tocopherols and tocotrienols in SkM is missing, whereas the existence of a relationship between irisin and Vit E has been demonstrated in a few studies on other tissues. Vit E, by regulating the level of irisin and other peptides in rats, protected the ovaries from ischemia-reperfusion injury [133]. However, the protective effect of the oral administration of an α-tocotrienol-rich fraction on obesity-induced glucose intolerance and inflammation in rats might be independent of irisin expression [134]. The regulation of irisin by the master muscle transcriptional regulator PGC-1α and the ability of Vit E to control its expression in other tissues allows one to speculate the potential role of Vit E in irisin modulation also in SkM. This could offer a strategy to facilitate recovery when normal exercise is not possible. Regarding myokines, sphingosine 1-phosphate S1P/S1P receptor (S1PR) signaling has been recently demonstrated to enhance the production and secretion of irisin and its effects on myoblast proliferation and differentiation [23].

### 4.2. Sphingolipids

#### 4.2.1. Metabolism and Functions in SkM

**Figure 3:**
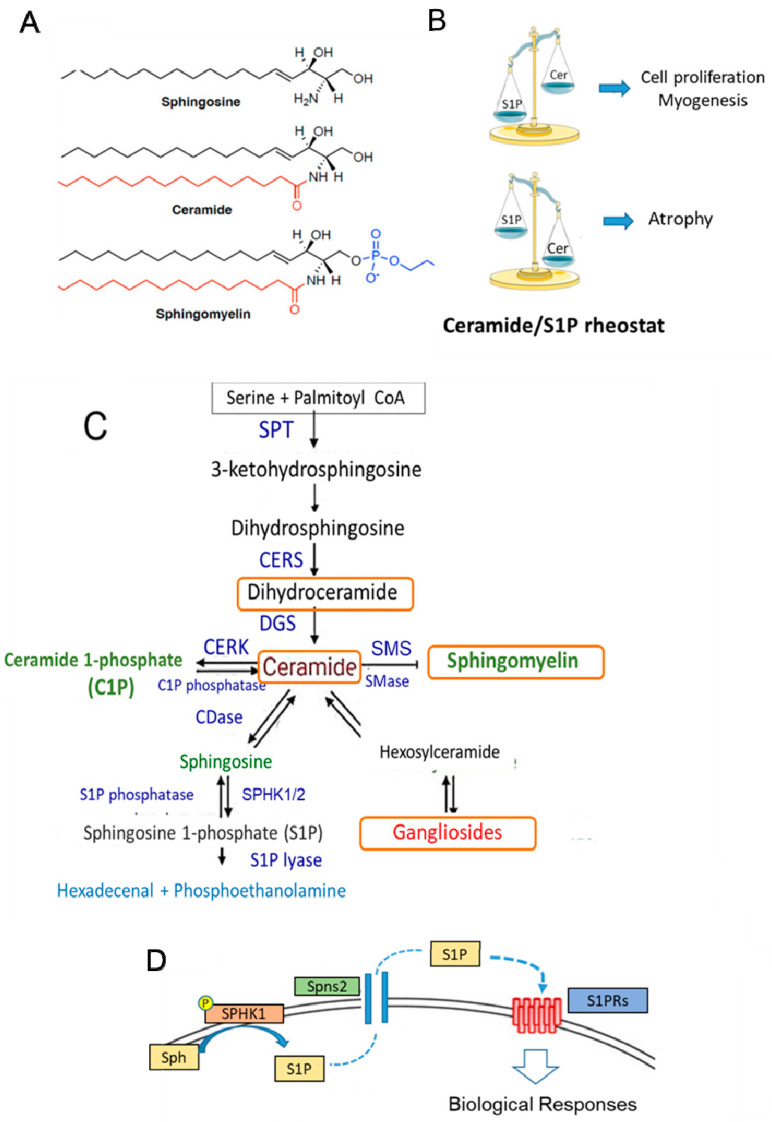
Sphingolipid structure, metabolism, and function in SkM. (**A**) The stereochemical structure of the sphingolipids sphingosine, ceramide, and sphingomyelin. (**B**) Balance between ceramide (Cer) and S1P content can affect the cellular fate. In SkM, S1P is a pro-survival and pro-myogenic factor, whereas ceramide inhibits myogenesis and promotes cell atrophy. (**C**) Sphingolipid metabolism. The de novo SL pathway occurs in the endoplasmic reticulum, where the condensation of serine and palmitoyl CoA by serine palmitoyltransferase (SPT) generates ceramide, which is then shuttled to the Golgi apparatus. Here, it is used as the building structure for the synthesis of sphingomyelin and other complex sphingolipids. Cer can also be generated by sphingomyelin hydrolysis catalyzed by sphingomyelinase (SMase) activity. Ceramide is then converted reversibly to sphingosine by ceramidase (CDase) or phosphorylated to ceramide-1-phosphate (C1P) by ceramide kinase (CERK) activity. Successively, sphingosine is phosphorylated by two isoforms of sphingosine kinases, SPHK1 and SPHK2, to S1P. The exit from the sphingolipid synthesis pathways occurs through S1P lyase, which promotes the degradation of S1P into hexadecenal and phosphoethanolamine. The latter is further metabolized into palmitoyl CoA. S1P is also a substrate of specific S1P phosphatases, which generate sphingosine. CERS: ceramide synthase; DEGS: sphingolipid delta 4-desaturases; SMS: sphingomyelin synthase; KDS: 3-ketodihydrosphingosine reductase. (**D**) S1P produced by the active membrane-bound SPHK from sphingosine (Sph) can be transported outside the cell by an ATP-binding cassette transporter named spinster homolog 2 (Spns2) and, acting as a ligand for specific GTP-binding protein-coupled receptors (S1PRs), can affect different signaling pathways. The orange boxes indicate the metabolites that are affected by Vit E.

Sphingolipids (SLs), structural components of the membranes of all eukaryotic cells, are also bioactive molecules characterized by the presence of sphingoid bases: a sphingosine backbone linked to one hydrophobic acyl chain and a phosphate head group ester (Figure 3A). SLs participate in the control of a variety of important cell functions, such as cell growth, differentiation, inflammation, senescence, and apoptosis [135,136,137,138,139] (Figure 3B–D). In particular, S1P can act as an intracellular mediator and, after being transported outside the cell, as a ligand for specific heterotrimeric GTP-binding protein-coupled receptors, named S1PRs [135,136,137], which are widely expressed in almost all cell types. Several studies have reported the crucial role of ceramide and S1P in SkM cell biology [140,141,142,143]. Similar to other tissues, ceramide and S1P act in an opposite manner (Figure 3B). In particular, reduced production of S1P by silencing SPHK activity enhances the proliferation of myoblasts and delays myogenesis [141,143], while ceramide induces cell growth arrest. In mature differentiated SkM cells, the reduced content of active SPHK and high level of ceramide promote cell atrophy, as observed in in vitro cells as well as in SkM tissue obtained from cachectic mice models [143,144]. It is worth noting that the SPHK1/S1P axis exerts a protective action on denervated SkM [145] or damaged muscle fibers [146]. Most of these effects are likely associated with the capability of SLs to maintain cellular redox homeostasis by controlling NADPH oxidase, mitochondrial integrity, and antioxidant enzymes [147,148]. Several studies also support the role of the S1P/S1PR axis in the control of mechanical inputs (i.e., extracellular matrix stiffness) and cytoskeleton remodeling [149,150]. During senescence, the increase in dipeptidyl peptidase 4 (DPP4/CD26) expression [151] is required for the senescence-associated secretory phenotype and activation of galactosidase expression and specific signaling, such as src/p38MAPK/NFkB. Although the role of SLs in aging has not been fully investigated, they can affect all these signaling pathways [147,148,152], and thus, SLs may modulate the senescent phenotype in SkM cells. Moreover, recent findings indicate that alterations in gene and protein expression in SL metabolism (i.e., CERS1 and DEGS1) are linked to age-related impairments [153,154].

#### 4.2.2. Sphingolipids and Vit E

Increasing evidence supports a correlation between a low level of Vit E and the level of SLs in the plasma and pathological conditions, such as inflammation and cancer. In particular, it has been reported that γ-tocopherols and tocotrienols have anti-cancer effects through the control of several key mediators (nicely reviewed in [155]), including specific SLs. Moreover, lipidomic studies indicate that γ-tocotrienols alter lipid metabolism (ceramide synthesis) during inflammation in LPS-primed bone marrow-derived macrophages [156]. Moreover, a decrease in Vit E content in pro-oxidant conditions is correlated with the accumulation of ceramide and changes in SLs in liver cells [157]. Regarding SkM, firstly, Albarracin et al. [158] compared SkM tissues from Vit E-deficient and control rabbits and reported that low levels of Vit E lead to an increase in lipids (i.e., gangliosides, sphingomyelin, and neutral glycosylceramides). Alpha-tocopherol can prevent the apoptosis promoted by 7-ketocholesterol in A7R5 smooth muscle cells, and, notably, 7-ketocholesterol, a pro-atherogenic compound, is able to alter SLs in the raft domains of the cell membrane [159], supporting the potential regulation of SLs by α-tocopherol. It can be speculated that in SkM, tocopherols and tocotrienols, acting as antioxidants, membrane stabilizers, and signaling mediators, can differently activate SL metabolism, and their intracellular re-localization may lead to a control of SkM biology. Moreover, it would be of interest to study this functional relationship also in specific SkM dysfunctions, such as those related to disuse, aging, and microgravity.

## 5. Conclusions

Since the clinical treatment of many SkM dysfunctions is still a huge challenge, the prevention of SkM degeneration is the only possibility in many circumstances. SkM is a tissue sensitive to reduced levels of α-tocopherol [17,27], and the beneficial effects of Vit E supplementation on SkM health have already been reported in some animal and human studies. Rigorous protocols using well-defined dosages and times, specific Vit E forms, chemically characterized extracts, and/or a combination of tocopherols and tocotrienols should be set up in order to ascertain whether and in which conditions Vit E has positive effects. Myokines and bioactive SLs, in particular, S1P and ceramide, could be new potential targets/effectors of tocopherols and tocotrienols. The identification of Vit E signaling mediators and specific and dynamic molecular/functional interactions may help to better define the potential use of tocopherols and tocotrienols in counteracting SkM dysfunctions.
